# Awareness and Risk Assessment of Breast Cancer Among Women in Saudi Arabia: A Cross-Sectional Study

**DOI:** 10.7759/cureus.51450

**Published:** 2024-01-01

**Authors:** Faisal Alnaqrani, Mohammed J Almuayrifi, Lama S Alhumaidan, Amer S Alsaeri, Abdulrahman M Alfantoukh, Rola M Alradaddi

**Affiliations:** 1 General Surgery, Taibah University, Medina, SAU; 2 College of Medicine, Taibah University, Medina, SAU; 3 College of Medicine, Unaizah College of Medicine and Medical Sciences, Qassim University, Unaizah, SAU; 4 College of Medicine, Majmaah University, Al Majma'ah, SAU; 5 College of Medicine, Imam Mohammad Ibn Saud Islamic University, Riyadh, SAU

**Keywords:** saudi arabia, gail model, breast cancer, risk assessment, awareness

## Abstract

Background

Breast cancer is the most common female cancer worldwide including in Saudi Arabia. As a result, many cases are diagnosed at an advanced stage, leading to a poor outcome. Understanding risk perception is a significant component of awareness of breast cancer risks. It can be helpful to reduce the mortality of breast cancer via increasing awareness of the risk factors.

Objective

Our study was designed to assess the level of awareness among women in Saudi Arabia regarding breast cancer, including knowledge about risk factors, symptoms, and the importance of early detection.

Methods

A cross-sectional study was conducted, and participants were randomly selected. The target population in this study is all women in Saudi Arabia. Data was collected via an online questionnaire. The collected data was analyzed using the Statistical Package for the Social Sciences (IBM SPSS Statistics for Windows, IBM Corp., Version 21.0, Armonk, NY).

Results

About 713 women were enrolled in this study. Most of them (69%) were within the age group of 35-45 years old. Age at menarche was found to be 12-13 years old in 313 (43.9%) of the participants and age at first live birth was found to be 20-24 in about 360 (50.5%). The number of population with first-degree relatives that have a history of breast cancer is one relative in 126 (17.7%) of the participants and about 36 (5%) had breast biopsy. About 76 (10.7%) of the participants were considered as having a high risk of breast cancer according to the estimated five-year breast cancer-risk assessment (had a five-year breast cancer risk >1.66%). The mean knowledge score of the participants about breast cancer was found to be 4.62 ± 1.86 out of 8. About 509 (71.4%) of the participants were considered to be having a good level of knowledge about breast cancer. Long-term hormone contraceptive use and older age can increase the chance of developing breast cancer as agreed on by 363 (50.9%) and 287 (40.3%) of the participants respectively. A total of 677 (95%) of the participants mentioned that early detection is important to survive breast cancer. And 639 (89.6%) of the participants think that breast cancer is treatable and about 288 (40.4%) think that the suitable age to start mammography is above 30 years old.

Conclusion

There is a good general knowledge and awareness about breast cancer among study participants. There were few knowledge gaps regarding the effect of obesity, hormonal contraceptives and older age on the association with breast cancer. About 10% of the participants were found to be having high five-year breast cancer risk.

## Introduction

Breast cancer is a major global health concern, constituting about 25% of all cancer cases and being the primary cause of cancer-related mortality [1]. It is the most commonly diagnosed cancer among women, accounting for roughly 75% of all cases. In Saudi Arabia, breast cancer is the most prevalent cancer in women, making up 29% of female cancer cases, with its incidence on the rise [2]. Early detection, precise risk assessment, and prompt treatment are vital to improving survival rates and reducing the burden of breast cancer. Mammography is considered the gold standard for screening, contributing to lower breast cancer mortality [3]. However, in Saudi Arabia, mammography use remains suboptimal due to limited access, cultural beliefs, and insufficient knowledge about breast cancer risk factors, symptoms, and early detection importance [4]. Enhancing public awareness, promoting routine screening, and identifying high-risk individuals are critical to improving early diagnosis and treatment outcomes. Several studies [5,6] have examined breast cancer awareness, risk perception, and screening practices among Saudi women. Yet, more comprehensive research is needed to gain a comprehensive understanding of breast cancer awareness and risk assessment nationwide. This research would facilitate the development of tailored interventions and public health strategies to address the unique needs and challenges of Saudi women. Risk assessment tools such as the Gail model (GM) and the Breast Cancer Risk Assessment Tool (BCRAT) are available to estimate an individual's breast cancer risk and enable targeted interventions and personalized prevention strategies [7,8]. However, their limited use in Saudi Arabia and their suitability for the Saudi population require further investigation [9]. The aim of this cross-sectional study is to assess breast cancer awareness and risk perception among a diverse and representative sample of Saudi Arabian women. Additionally, the study will evaluate the applicability and accuracy of existing breast cancer risk assessment tools in identifying high-risk Saudi Arabian women. These findings will guide the development of culturally sensitive and effective interventions to enhance breast cancer screening rates and prevention efforts in Saudi Arabia.

Breast cancer is a prevalent global health issue, affecting about 36% of all cancer patients in 2018 [10,11]. Approximately 2.089 million women were diagnosed with breast cancer that year. Although breast cancer incidence is rising worldwide, developed countries have the highest rates, accounting for nearly half of all cases. The etiology is multifactorial and these factors include gender, age, and a country's economic status, as suggested by epidemiological data. Hormonal factors, such as estrogen (ER) exposure, and reproductive factors like the number of children, age at first childbirth, and breastfeeding, also play a significant role. Genetic predisposition (e.g., BRCA1/BRCA2 mutations), hormone replacement therapy, an unhealthy diet leading to obesity, and certain behaviors like hormonal contraception, alcohol consumption, and early exposure to ionizing radiation are also considered significant risk factors for breast cancer [12,13]. The majority of breast cancer cases involve adenocarcinomas, with invasive ductal carcinoma and less severe invasive lobular carcinoma being the primary forms [14]. Typically, breast cancer is detected during routine mammography screenings, particularly recommended for women over 50 years old. If mammography identifies abnormalities or lumps, further radiographic evaluation is necessary. In cases of suspected malignancy or inconclusive results, a biopsy is conducted, and the tissue is examined through histopathology. The status of axillary lymph nodes is determined through clinical examination and biopsy of suspicious nodes [15-17]. Treatment for breast cancer is personalized based on factors like grade, stage, and molecular subtype. Grade assesses the appearance of tumor cells, while stage assesses cancer's extent, considering factors such as tumor size, lymph node status, and the presence of metastases. Non-metastatic breast cancer is treated with a combination of surgery, radiotherapy, chemotherapy, targeted therapy, and hormone therapy depending on clinical and pathologic features. Treatment of patients with metastatic breast cancer aims to control tumor spread using systemic therapies. Challenges include treatment resistance and recurrence, with around 10-20% of early-stage cases experiencing recurrent disease, primarily as metastases. Developing targeted therapies for specific breast cancer subgroups is crucial. Hormone therapy (e.g., tamoxifen) is used for tumors positive for ER, and targeted therapy (e.g., trastuzumab) is administered to those with human epidermal growth factor receptor 2 (HER2) positive tumors. Key prognostic factors impacting patient outcomes include cancer stage and receptor status. Women at higher risk of breast cancer, such as those with a positive BRCA mutation, may be advised to consider preventive measures like bilateral prophylactic mastectomy [18,19].

Objective

To assess the level of awareness among women in Saudi Arabia regarding breast cancer, including knowledge about risk factors, symptoms, and the importance of early detection, and to validate and adapt existing breast cancer risk assessment tools, such as the GM and the BCRAT, for the Saudi population.

## Materials and methods

Study design

It was a cross-sectional study with IRB number TU-040-22 from Taibah University, College of Medicine, Research Ethics Committee (CM-REC).

Study area and setting

The research was conducted in Saudi Arabia. An online form designed using Google Forms and was sent via WhatsApp, and then it was distributed electronically via social media apps, each co-author took responsibility for delivering the questionnaire to a specific area in Saudi Arabia ensuring that all populations were covered.

Study population and sampling

The research proposal started in Saudi Arabia in the year 2023 in February and was accepted by Institutional Review Boards in June data was collected directly after that via an online questionnaire that was sent via WhatsApp. Special criteria included females who live in Saudi Arabia, 35 and older (as in the GM), and excluded those who declined to participate in the study, males, and females not living in Saudi Arabia.

Sample size and selection of sample

The sample size was estimated by an online sample size calculator (Raosoft, Raosoft, Inc., Seattle, WA) [20] and by using a margin of error of 5% and a confidence interval of 95% assuming an average response for most of the questions of 50%. According to the population of 34110821 in Saudi Arabia, the required sample size was 385. The questionnaire was distributed online, and it included all the subjects who matched the inclusion criteria and agreed to participate at the time of data collection and excluded any subjects who fulfilled the exclusion criteria.

Methods of gathering data

The research utilized a structured questionnaire, which was formulated by the authors Erbil N, Dundar N, Inan C, and Bolukbas N [21].

List of variables

The questionnaire form and projected breast cancer risk (calculated risk) were determined using the modified GM. The questionnaire form obtained information about the women’s socio-demographic characteristics age, education level, occupation, social security, family income, marital status, place of residence, woman’s husband's education level and occupation, and other factors related to breast cancer ages of menarche and first childbirth, having a family history of breast cancer, having a biopsy.

Research instrument (questionnaire) and its validation

The questionnaire is a validated questionnaire that has previously been published using the National Cancer Institute’s online BCRA or the Gail Risk Assessment Tool [8].

Data analysis

The analysis was conducted using the Statistical Package for the Social Sciences (IBM SPSS Statistics for Windows, IBM Corp., Version 21.0, Armonk, NY) and was based on the types of variables and differential statistics in the form of the chi-square test. The significance of the data was determined using the p-value. Data was presented in the form of tables and graphs using descriptive statistics.

Ethical consideration

The research has been approved by Taibah University, CM-REC, with reference number: TU-040-22. Participation was voluntary, and participants were permitted to withhold their consent to participate in the study. All data from the questionnaire was kept confidential, and only researchers can access participants' information.

## Results

Characteristics of the participants

A total of 713 women were included in this study. Most of them 492 (69%) were within the age group of 35-45 years old. Regarding educational level, the majority (75.3%) of the participants had a university educational level. More than half (53.4%) of the participants were government sector employees, 288 (40.4%) were housewives and 44 (6.2%) were private sector employees. Husband's educational level was found to be university level for about 372 (52.2%) of the participants. Five hundred and ninety-nine (84%) were having middle average family income (Table 1).

**Table 1:** Socio-demographic characteristics of the study participants (n=713)

Variable	Categories	Frequency	Percent
Age (in years)	35-45	492	69
	46-55	195	27.3
	56 or more	26	3.6
Education level	Primary school	23	3.2
	Secondary school	25	3.5
	High school	128	18
	University	537	75.3
Occupation	Housewife	288	40.4
	A government sector employee	381	53.4
	A private-sector employee	44	6.2
Husband’s education level	Primary school	38	5.3
	Secondary school	54	7.6
	High school	158	22.2
	University	372	52.2
	Unmarried	91	12.8
Average family income	High	77	10.8
	Middle	599	84
	Low	37	5.2

**Figure 1:**
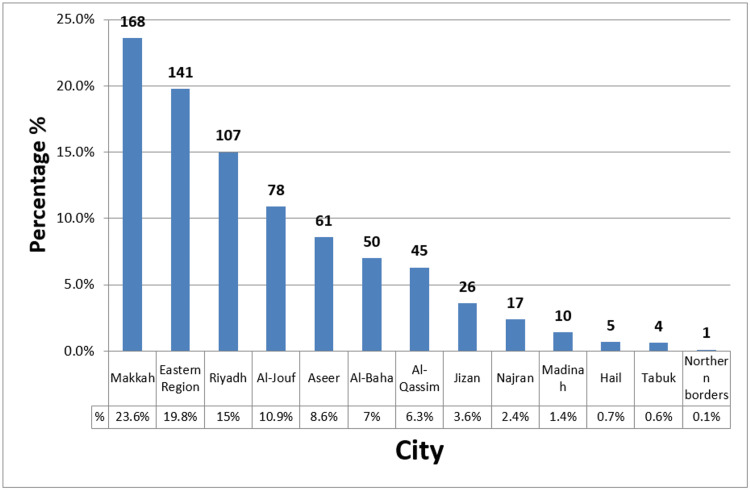
Place of residence of the study participants

Most of the participants were from Makkah and the eastern region (23.6% and 19.8% respectively) followed by Riyadh which represents the residence of 107 (15%) of the participants and the rest were from other regions (Figure 1).

Risk factors for the BRCA tool of women

Age at menarche was found to be 12-13 years old in 313 (43.9%) of the participants and more than 14 years old in 206 (28.9%) of the participants. Age at first live birth was found to be 20-24 in 360 (50.5%) of the participants. The number of first-degree relatives with breast cancer was one relative in 126 (17.7%) of the participants and more than one relative in 53 (7.4%) of the participants. The rest were with no relatives having breast cancer or unknown 36 (5%) of the participants had breast biopsy (Table 2).

**Table 2:** The risk factors for breast cancer among women in Saudi Arabia

Variable	Categories	Frequency	Percent
Age at menarche (years)	Unknown	99	13.9
	07-Nov	95	13.3
	Dec-13	313	43.9
	≥ 14	206	28.9
Age at first live birth (years)	No children	89	12.5
	≤ 20	155	21.7
	20-24	360	50.5
	≥ 30	109	15.3
Number of first-degree relatives with breast cancer?	Unknown	176	24.7
	Zero relatives	358	50.2
	One relative	126	17.7
	More than one	53	7.4
Did you do a breast biopsy?	Yes	36	5
	No	668	93.7
	Do not know	9	1.3

About 76 (10.7%) of the participants were considered as having a high risk of breast cancer according to the estimated five-year breast cancer-risk assessment (had a five-year breast cancer risk >1.66%). The mean five-year risk of the participants was 0.96 ± 0.80 with a minimum risk of 0.2 and maximum risk of 7.6 and the mean risk of participants up to age 90 years was 12.27 ± 7.07 with a minimum risk of 2.2 and maximum risk of 40.2 (Table 3).

**Table 3:** Mean risk values for five-year risk and mean risk values up to age 90 years of participants according to the BRCA tool (n=713) * had a five-year breast cancer risk >1.66% BRCA Tool: Breast Cancer Risk Assessment Tool

Risk	Mean (%)	Standard Deviation	Minimum Risk	Maximum Risk
Mean five-year risk of participants	0.96	0.80	0.2	7.6
Mean five-year risk for women at high risk*	2.74	1.20	1.7	7.6
Mean risk of participants up to age 90 years	12.27	7.07	2.2	40.2
Mean risk up to age 90 years for women at high risk*	23.35	10.64	3.1	40.1

The mean knowledge score of the participants about breast cancer was found to be 4.62 ± 1.86 (range 0-8) out of 8. A total of 509 (71.4%) of the participants were considered to be having a good level of knowledge about breast cancer (i.e. scored 4 or more out of 8) while 204 (28.6%) of the participants were found to be having poor knowledge.

Older age can increase the chance of developing breast cancer as agreed on by 287 (40.3%) of the participants, 184 (25.8%) responded negatively and 242 (33.9%) didn't know. Three hundred and fifty-six (49.9%) of the participants think that having a close relative with breast cancer can increase the chance of developing breast cancer. Three hundred and sixty-three (50.9%) of the participants agreed that long-term hormone contraceptive use can increase the chance of developing breast cancer. Six hundred and seventy-seven (95%) of the participants mentioned that early detection is important to survive breast cancer. Obesity and lack of exercise were considered risk factors for breast cancer in 325 (45.6%) of the participants. Six hundred and thirty-nine (89.6%) of the participants think that breast cancer is treatable (Table 4).

**Table 4:** Knowledge of breast cancer among women in Saudi Arabia

Variable	Yes	No	Don't Know
	n (%)		
Do you think that being older can increase the chance of developing breast cancer?	287 (40.3)	184 (25.8)	242 (33.9)
Do you think that not breastfeeding can increase the chance of developing breast cancer?	372 (52.2)	164 (23)	177 (24.8)
Do you think that having a close relative with breast cancer can increase the chance of developing breast cancer?	356 (49.9)	203 (28.5)	154 (21.6)
Do you think that long-term hormone contraceptive use can increase the chance of developing breast cancer?	363 (50.9)	114 (16)	236 (33.1)
Do you think that early detection is important to surviving breast cancer?	677 (95)	12 (1.7)	24 (3.4)
Do you think that obesity and lack of exercise are considered a risk for breast cancer?	325 (45.6)	149 (20.9)	239 (33.5)
Do you think that it is possible to treat breast cancer?	639 (89.6)	9 (1.3)	65 (9.1)

**Figure 2:**
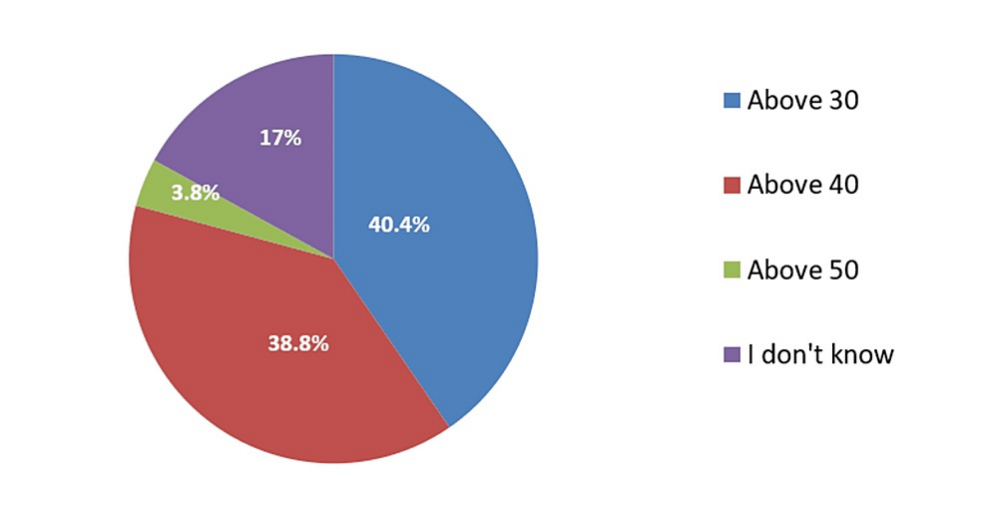
Participants' perception of the suitable age for beginning mammography

A total of 288 (40.4%) of the participants think that the suitable age to start mammography is above 30 years old, 277 (38.8%) reported that the suitable age for mammography is above 40 years old, and 27 (3.8%) mentioned above 50 years old as the suitable age for mammography whereas 121 (17%) didn't know (Figure 2).

## Discussion

General knowledge and awareness about breast cancer and its risk assessment are important as good knowledge about these factors could result in a reduced prevalence of breast cancer in the community. Some factors are modifiable and others are non-modifiable [22].

More than two-thirds (69%) of the participants were within the age group of 35-45 years old. In regards to educational level, the majority (75.3%) of the participants were having university educational level. Most of the participants 168 (23.6%) were from Makkah and the eastern region (19.8%) of the participants. Participants' age at menarche was found to be 12-13 years old in about less than half (43.9%) of the participants and more than 14 years old in (28.9%) of the participants, similar findings were reported in the parallel study which found that the mean age at menarche was found to be 12.4 years old [23]. Age at first live birth was found to be 20-24 in about half (50.5%) of the participants analogous findings were reported in a congruent study carried out by Talukder et al. in which the mean maternal age at first life birth was found to be 22.38 years old [24]. Only 5% of the participants had a breast biopsy. Ten percent (10.7%) of the participants were considered as having a high risk of breast cancer according to the estimated five-year breast cancer risk assessment, this percentage is lower than that mentioned in a study carried out in Iran in which a five-year cancer risk assessment was found to be high in (16.2%) of the participants [25]. Concerning knowledge and awareness assessment out of a total score of 8, the mean knowledge score of the participants about breast cancer was found to be 4.62. More than two-thirds (71.4%) of the participants were considered to be having a good level of knowledge about breast cancer while 28.6% of the participants were found to be having poor knowledge of the current level of knowledge demonstrated in the current study was found to be higher than which reported in a parallel study in which moderate amount of knowledge was reported, this could be attributed to different factors related to the studied samples [26].

An increase in age (old age) can increase the chance of developing breast cancer as agreed on by (40.3%) of the participants. Older age increases the chance of developing breast cancer as mentioned in the study conducted in the Polish study [27]. Decreased or no breastfeeding could increase the chance of developing breast cancer as believed by more than half (52.2%) of the participants. Breastfeeding was reported to reduce the risk of developing breast cancer as reported in the study [28]. Half (50.9%) of the participants agreed that long-term hormone contraceptive use can increase the chance of developing breast cancer. Contraceptive use could increase breast cancer risk by 20% as reported in the congruent study [29]. Obesity and lack of exercise are considered risk factors for breast cancer which are less than half (45.6%) of the participants. Less than half (40.4%) of the participants think that the suitable age to start mammography is above 30 years old, 38.8% reported that the suitable age for mammography is above 40 years old, and 3.8% mentioned above 50 years old as the suitable age for mammography whereas 17% don't know suitable age for screening with mammography that was mentioned in the parallel study conducted by Løberg et al. in which age of 40 up to 74 was mentioned for screening mammography [30].

Limitations

The conservative nature of Saudi society posed obstacles in assembling our research sample, particularly due to the sensitive nature of discussing breast cancer. Engaging in conversations about such health issues proved challenging. Moreover, we experienced difficulties in reaching the specific sample we aimed for, further complicating the research process.

## Conclusions

Good general knowledge and awareness about breast cancer among the study participants was observed. There were few knowledge gaps regarding the effect of obesity, hormonal contraceptives, and older age on developing breast cancer. About 10% of the participants were found to be having a high five-year breast cancer risk. These findings underscore the importance of targeted educational efforts to address knowledge gaps and promote early detection and prevention strategies among at-risk individuals.

## Appendices

Questionnaire

Socio-Demographic Data

1)      Education level?

a.       Primary school

b.      Secondary school

c.       High school

d.      University

2)      Occupation?

a.       Housewife

b.      A government sector employee

c.       A private-sector employee

3)      Husband’s education level?

a.       Primary school

b.      Secondary school

c.       High school

d.      University

4)      Average Family income?

a.       High

b.      Middle

c.       Low

5)      Place of residence?

a.       City

b.      Village

Risk Factors for the BRCA Tool of Women

6)      Age?

a.       ≤45-Year-old

b.      46-55-Year-old

c.       ≥55-Year-old

7)      Age at menarche (years)?

a.       Unknown

b.      7-11-Year-old

c.       12-13-Year-old

d.      ≥14-Year-old

8)      Age at first live birth (years)?

a.       No children

b.      ≤20-Year-old

c.       20-24-Year-old

d.      ≥30-Year-old

9)      Number of first-degree relatives with breast cancer?

a.       Unknown

b.      Zero relatives

c.       One relative

d.      More than one

10)   Did you do a breast biopsy?

a.       Yes

b.      No

c.       Do not know

Knowledge Section

11)  Do you think that being older can increase the chance of developing breast cancer?

a.       Disagree

b.      Agree

c.       Not Sure

12)   Do you think that not breastfeeding can increase the chance of developing breast cancer?

a.       Disagree

b.      Agree

c.       Not Sure

13)  Do you think that having a close relative with breast cancer can increase the chance of developing breast cancer?

a.       Disagree

b.      Agree

c.       Not Sure

14)   Do you think that long-term hormone contraceptive use can increase the chance of developing breast cancer?

a.       Disagree

b.      Agree

c.       Not Sure

15)   Do you think that early detection is important to surviving breast cancer?

a.       Disagree

b.      Agree

c.       Not Sure

16)   Do you think that obesity and lack of exercise are considered a risk for breast cancer?

a.       Disagree

b.      Agree

c.       Not Sure

17)  Do you think that it is possible to treat breast cancer?

a.       Disagree

b.      Agree

c.       Not Sure
